# Multiplexed engineering and precision gene editing in cellular immunotherapy

**DOI:** 10.3389/fimmu.2022.1063303

**Published:** 2022-11-22

**Authors:** Alexander Biederstädt, Gohar Shahwar Manzar, May Daher

**Affiliations:** ^1^ Department of Stem Cell Transplantation and Cellular Therapy, The University of Texas MD Anderson Cancer Center, Houston, TX, United States; ^2^ Department of Medicine III, Hematology and Oncology, School of Medicine, Technical University of Munich, Munich, Germany; ^3^ Department of Radiation Oncology, The University of Texas MD Anderson Cancer Center, Houston, TX, United States

**Keywords:** cell engineering, gene editing, immune effector cell, cell therapy, CAR (chimeric antigen receptor), CRISPR screening

## Abstract

The advent of cellular immunotherapy in the clinic has entirely redrawn the treatment landscape for a growing number of human cancers. Genetically reprogrammed immune cells, including chimeric antigen receptor (CAR)-modified immune effector cells as well as T cell receptor (TCR) therapy, have demonstrated remarkable responses across different hard-to-treat patient populations. While these novel treatment options have had tremendous success in providing long-term remissions for a considerable fraction of treated patients, a number of challenges remain. Limited *in vivo* persistence and functional exhaustion of infused immune cells as well as tumor immune escape and on-target off-tumor toxicities are just some examples of the challenges which restrain the potency of today’s genetically engineered cell products. Multiple engineering strategies are being explored to tackle these challenges.The advent of multiplexed precision genome editing has in recent years provided a flexible and highly modular toolkit to specifically address some of these challenges by targeted genetic interventions. This class of next-generation cellular therapeutics aims to endow engineered immune cells with enhanced functionality and shield them from immunosuppressive cues arising from intrinsic immune checkpoints as well as the hostile tumor microenvironment (TME). Previous efforts to introduce additional genetic modifications into immune cells have in large parts focused on nuclease-based tools like the CRISPR/Cas9 system or TALEN. However, nuclease-inactive platforms including base and prime editors have recently emerged and promise a potentially safer route to rewriting genetic sequences and introducing large segments of transgenic DNA without inducing double-strand breaks (DSBs). In this review, we discuss how these two exciting and emerging fields—cellular immunotherapy and precision genome editing—have co-evolved to enable a dramatic expansion in the possibilities to engineer personalized anti-cancer treatments. We will lay out how various engineering strategies in addition to nuclease-dependent and nuclease-inactive precision genome editing toolkits are increasingly being applied to overcome today’s limitations to build more potent cellular therapeutics. We will reflect on how novel information-rich unbiased discovery approaches are continuously deepening our understanding of fundamental mechanisms governing tumor biology. We will conclude with a perspective of how multiplexed-engineered and gene edited cell products may upend today’s treatment paradigms as they evolve into the next generation of more potent cellular immunotherapies.

## 1 Introduction

Chimeric antigen receptor (CAR) engineering of immune effector cells (IEC), such as T cells or Natural Killer (NK) cells, is a promising frontier of personalized cancer therapy, manifesting sustained remissions in specific populations of patients with relapsed or refractory (R/R) lymphoid malignancies (1). This approach entails reprogramming immune effector cells with synthetic receptors equipped with multiprong features enabling antigen recognition, downstream signaling, and costimulatory activation, ultimately resulting in targeted elimination of antigen-expressing tumor cells. The promise of CAR-T cell therapy has primarily been realized through CD19-directed therapies against B cell malignancies (2–6), and more recently through BCMA-directed therapies against multiple myeloma (7, 8), for which there are several commercial products now available. This success has spurred extensive preclinical investigation of cellular immunotherapy against other malignancies with an expectant dynamic and exciting pace of new indications. However, the clinical efficacy of CAR-based therapies against other malignancies, particularly solid tumors, has not yet been matched in many settings due to issues relating to trafficking, infiltration, clonal heterogeneity, off-target activity or on-target off-tumor activity against normal tissues, and the hostile immunosuppressive TME (9). There are additional logistical and clinical limitations to CAR-T cell therapy. The cumbersome and lengthy production associated with manufacturing an autologous product renders high expenditure and restrictive use (10). Even if previously generated allogeneic T cell products are HLA-matched with the recipient, genetic modification is still required to reduce the risk of graft-versus-host disease (GvHD) mediated by the native αβ T-cell receptor (TCR). Furthermore, CAR-T cell therapy can cause toxicities, such as immune effector cell–associated neurotoxicity syndrome (ICANS) and cytokine release syndrome (CRS), which can increase hospitalization duration and treatment cost (10). NK cells are innate immune cells that serve as an attractive alternative platform for CAR engineering and offer several advantages to the use of T cells, including avoiding the risk of GvHD, scalability in an off-the-shelf allogeneic cellular product, and a superior safety profile (10).

Enduring challenges with CAR engineered effector cells that may yield suboptimal response and disease relapse include i) antigen negative recurrence from immunoediting and antigen escape in the tumor, or ii) limited *in vivo* persistence or functional exhaustion of the CAR-engineered cells, resulting in antigen positive relapse (11). Strategies to combat antigen negative relapse thematically involve targeting multiple antigens either simultaneously or in a sequential manner. Approaches to overcome effector exhaustion or to increase proliferation and potency are varied and include i) altering the choice of co-stimulation (12), ii) cytokine cargo coupling (e.g. IL-15, IL-12, IL-18) with awareness of the detriment from unconstrained tonic signaling (13), and iii) synergizing CAR adoptive cell therapy with checkpoint blockade (14), among other tactics. Such advancements are being supplanted with pioneering, multiplexed gene editing tools including Clustered Regularly Interspaced Short Palindromic Repeats (CRISPR) based editing to introduce deliberate genetic perturbations that enhance the potency of CAR effectors by, for example, reprograming immune cell metabolism, equipping cells with methods to resist exhaustion, disrupting pathways for immunosuppression, or improving survival (11). Such interventions and investigations show enormous promise in the marriage of sophisticated, hypothesis-driven modification of immune cells in a site-directed fashion to modulate cellular pathways that negatively impact the efficacy and fitness of CAR-modified effector cells.

In this review, through the prism of challenges the field has been facing with CAR-based immunotherapeutic strategies, we will discuss the evolution of innovative multiplex engineering strategies and precision gene editing tools that are designed to enhance the therapeutic scope and potency of this groundbreaking personalized therapy.

## 2 Multiplexed engineering as a tool to enhance cellular therapies

The expanding possibilities in personalized medicine coincide with the dramatic improvements in immune cell engineering, most notably the successful transition of CAR-T and CAR-NK cells as well as TCR gene therapy (15) into the clinic.

One of the most valuable features of cellular immunotherapy lies in the capacity of infused immune cells to expand and evolve *in vivo*. Indeed, some studies point towards peak CAR transcript levels as assessed in the patients’ peripheral blood to be a potential predictor of clinical response (16, 17). Contrary to antibody-based strategies, which often need to be administered continuously to mitigate rapid *in vivo* degradation, cell therapy products are ‘living drugs’ which exhibit striking plasticity with regard to their phenotypic, transcriptomic and epigenetic composition post-infusion. This dramatic evolution is shaped by the continuous selective pressures arising from the interaction with both the tumor cells as well as bystander host immune cells and may have profound effects on the persistence, memory formation and anti-tumor potency of infused immune effector cells (18–20). Seminal studies have in recent years investigated the single-cell trajectories of infused CAR-T cells and found key pre-infusion transcriptomic signatures which correlate with response and potential toxicity and may in the future serve as predictive biomarkers to help guide data-driven treatment decisions (21–24).

Novel high-dimensional assays with single-cell resolution have recently elucidated the complex epigenetic underpinnings of T cell exhaustion (25–30), and it is likely that other immune effector cells, including genetically engineered lymphocytes are subject to and governed by many of the same mechanisms although much is still to be learned in this regard.

**Figure 1:**
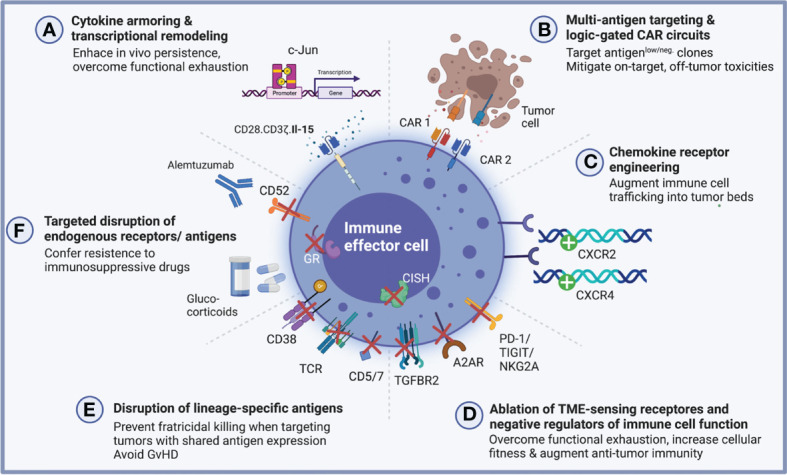
Multiplexed engineering and gene editing strategies to augment anti-tumor potency and cellular fitness. A growing number of multiplexed engineering approaches have been pursued to enhance the cellular fitness and augment anti-tumor potency of genetically engineered antigen-specific immune effector cells. **(A)** Cytokine-armored immune effector cells are reprogrammed using a multi-cistronic vector system which endows them with antigen specificity and furthermore encodes for a specific cytokine to provide autocrine growth support for enhanced functionality. In the setting of CAR-NK cells, this strategy has successfully been employed to augment *in vivo* persistence through ectopic IL-15 secretion (31). Epigenetic modulation by targeted insertion of transcription factors may confer exhaustion resistance as has been shown for c-Jun in CAR-T cells (32). **(B)** Combinatorial targeting strategies including dual CARs promise to increase IEC potency against antigen-low tumor clones by recognizing multiple relevant target antigens (33, 34). When combined with logic-gated circuits, these multi-receptor engineering approaches may overcome on-target, off-tumor toxicities which might arise from shared antigen expression between tumor and healthy tissue (35–37). **(C)** Through the targeted insertion of chemokine receptors, immune effector cells can be rewired to traffic more effectively into tumor beds, a critical lever to engineer cell products which are capable of combatting solid tumors (38, 39). **(D)** Building on the clinical success of immune checkpoint blockade, efforts are underway to genetically disrupt known negative regulators of immune cell function including, for instance, PD-1, TIGIT and NKG2A as well as TME-sensing receptors which bind immunosuppressive metabolites including adenosine and TGFβ (40–56). **(E, F)** Deletion of lineage-specific antigens or endogenous receptors may render engineered immune cells resistant to fratricidal killing, which is crucially important when targeting malignancies with an overlapping surfaceome (57, 58). For allogeneic CAR-T cells in particular, elimination of the endogenous T cell receptor (TCR) prevents the deleterious effects of alloreactivity and GvHD induction and site-directed engineering strategies now accomplish both simultaneously - deletion of the endogenous TRAC locus and CAR delivery (59, 60). Lastly, genetic deletion of endogenous antigens and receptors may prove advantageous when co-administrating lymphodepleting and immunosuppressive drugs (61, 62). Created with BioRender.com.

Multiplexed gene engineering, ultimately, attempts to modulate these cellular signatures to enhance the safety and efficacy of cell therapies. By combining the effective delivery of synthetic receptors with additional cellular modifications brought about by the opportunities arising from precise gene editing, the next generation of multiplexed engineered cellular immunotherapies strives to enhance anti-tumor potency, increase *in vivo* persistence and overcome functional exhaustion by increasing cellular fitness and/or shielding engineered immune cells from the inhibitory cues received from endogenous checkpoints or immunosuppressive metabolites in the tumor microenvironment (TME) by introducing multiple genetic modifications (**Figure 1**).

In the following section, we will outline the most pivotal examples of multiplexed engineered immune cells, a growing fraction of which have moved into clinical testing.

### 2.1 Synthetic transgenic payloads for enhanced functionality

#### 2.1.1 Cytokine armoring

Cytokine armoring was one of the early examples for how multiplexed engineering can endow genetically modified immune cells with enhanced functionality (**Figure 1A**).

Bi- and multi-cistronic vector systems are now routinely deployed to deliver synthetic external receptors for tumor antigen recognition together with additional molecular payloads such as cytokines for autocrine growth support (31, 63–71). For NK cells in particular, IL-15 has long been known to be a critical determinant for prolonged *in vivo* persistence (71) and different strategies have been devised to endow NK cells with IL-15 mediated autocrine growth support. In a phase I/II trial led by our group, NK cells engineered to express a CD19-directed CAR molecule together with IL-15 demonstrated remarkable responses in heavily pre-treated patients with CD19 positive hematologic malignancies (16, 31). Similar strategies are being pursued by others, for instance by retaining IL-2 within the endoplasmic reticulum (72) or by equipping NK cells with a membrane-bound IL-15/IL-15 receptor fusion protein (73).

Building on the observation that exposure to certain cytokines can fundamentally remodel the transcriptomic signature of immune cells and enhance their potency and even induce formation of a memory-like phenotype (74–77), there has been a growing interest in exploring other cytokines beyond IL-15 to engineer more powerful immune cell immunotherapies for sustained clinical responses (13, 78). Interestingly, this transcriptional remodeling can give rise to novel, thus far unrecognized, immune cell signatures, as exemplified by a recent report on IL-12-transduced CAR-T cells which emulate an NK cell-like phenotype with the capacity for antigen-independent HLA-E-restricted killing – a property which might complement CAR-driven responses by preventing immune escape *via* antigen loss (78).

Other strategies employed to remodel the transcriptional landscape and tune CAR-modified immune cells towards higher anti-tumor potency rely on the direct ectopic expression of transcription factors as exemplified by work from Crystal Mackall’s group on c-Jun signaling to overcome CAR-T functional exhaustion (32).

#### 2.1.2 Multi-targeting CARs & rational design of logic circuits

CAR-engineered cellular immunotherapies have shown remarkable paradigm-shifting successes particularly for liquid cancers. CD19-positive lymphoid malignancies are particularly well-suited to be targeted using CAR-engineered IECs as target antigen expression is relatively high and on-target off-tumor toxicities can be managed in many cases. Although antigen-low or antigen-negative relapses represent a major challenge (79) and efforts are underway to fine-tune CAR signal strength to address low antigen density (80), other human cancers have been disproportionally harder to target as they lack uniform expression of tumor-specific antigens or are characterized by a higher degree of clonal heterogeneity. Solid tumors in particular pose extraordinary challenges to engineered immune cells as they exhibit a set of unique challenges including shared antigen expression with healthy tissues, impaired trafficking, and homing into tumor beds, a hostile and immunosuppressive tumor microenvironment (TME) and a more heterogenous clonal architecture. Overcoming these challenges, hence, promises to target a broader range of human cancers and has driven a spur of innovation in recent years.

One strategy to address the relative paucity of tumor-specific antigens as well as tumor immune evasion due to clonal evolution and emergence of antigen-negative clones has been to engineer bispecific dual or tandem CAR-modified immune effector cells, some of which are governed by logic-gated circuits to specifically differentiate between shared targets expressed on cancerous cells versus healthy tissues (**Figure 1B**).

Recent work on bispecific CD19/CD22-directed CAR-T cells demonstrated the potential of dual targeting approaches to enhance anti-tumor activity and efforts are underway to further advance multi-specific CAR targeting both in the pre-clinical and clinical settings (33, 34, 79, 81, 82). Advancements in multi-receptor-based engineering approaches and in the ability to reprogram the intracellular signaling networks underpinning CAR activation has allowed for the design of logic-gated CAR-T cells which can effectively target tumor-associated antigens while avoiding on-target off-tumor toxicity and thus sparing healthy tissues (83, 84). Preclinical data reported by the Seattle group have demonstrated how CAR-T cells targeting tumor-associated ROR-1 can be prevented from inducing otherwise toxic abrogation of healthy bone marrow stromal tissue by endowing them with synthetic notch receptors (SynNotch) which specifically recognize EpCAM or B7-H3 leading to tumor regression while sparing healthy tissues (35). In NK cells, similar strategies have been conceived, for instance the recent report of FLT3 OR CD33 NOT EMCN CAR-NK cells which are activated by CD33+ or FLT3+ leukemic blasts but inhibited by concomitant recognition of Endomucin, which is expressed by the majority of healthy hematopoietic progenitor cells (HSCs) (36).

In a similar vein, preliminary reports have laid out an IF-BETTER gated combinatorial CAR design which selectively targets AML bulk and leukemic stem cells using an ADGRE2-targeting CAR molecule combined with a CLECL12A-directed chimeric costimulatory receptor (CCR). By adding CLEC12A as a rationally selected combinatorial target, T cells engineered to express this logic-gated construct recognize both ADGRE2^high^/CLEC12A^neg^ and ADGRE2^low^/CLEC12A^high^ leukemic cells, preventing ADGRE2^low^ immune escape while sparing ADGRE2^low^/CLEC12A^neg^ healthy hematopoiesis (37). Efforts to further advance logic circuits for precision targeting of tumor cells are underway and allow the construction of various logical conditionalities (e.g., AND, OR, NOT). Today, these synthetic circuits are not restricted to extracellular antigens but can also be applied to recognize MHC-bound intracellular tumor-associated peptides, for instance *via* scFv fragments targeting specific peptide-MHC complexes fused to synNotch receptors for conditional activation (85).

Besides widening the therapeutic window when targeting shared antigens, dual CAR strategies can also be harnessed to mitigate fratricidal killing of neighboring CAR-engineered IECs, which can occur following transfer of the targeted antigen from the tumor cells to the IECs through means of trogocytosis. As was recently illustrated for CD19-directed CAR-NK cells, integration of KIR-based inhibitory CAR receptors can shield NK cells from such effects and yield more potent tumor killing (86).

Collectively, these early examples foreshadow the enormous potential of rationally designed multi-targeted CAR constructs to precisely target and discriminate tumor cells from normal cells, addressing one of the most foundational challenges in cancer therapy.

#### 2.1.3 Chemokine receptor engineering

Besides shared antigen expression, impaired trafficking of immune cells into tumors represents another formidable hurdle to overcome to engineer more effective cellular immunotherapies, particularly, when aiming to combat solid tumors. In the realm of CAR-T cells, many studies have evaluated multiplexed engineering strategies to equip CAR- and TCR-modified T cells with chemokine receptors for enhanced tumor infiltration (87–96). In NK cells, similarly, a number of studies have reported improved NK cell infiltration and anti-tumor potency by overexpressing chemokine receptors (38, 97, 98) or augmenting release of chemokines from tumor beds (99, 100). The first chemokine receptor-engineered CAR-NK cells to be reported leveraged ectopic CXCR4 expression for enhanced trafficking into glioblastoma tumor beds (39) (**Figure 1C**). Similar efforts are being pursued by others (101). Nevertheless, much is still to be learned to fully leverage the potential of augmented chemokine signaling as many challenges remain. Most noteworthy, the mechanisms of impaired tumor microcirculation resulting in inadequate chemotactic gradients as well as context-specific differences in the ultimate effects of chemokine receptor-ligand interactions need to be better understood for these tools to successfully transition into the clinic.

## 3 Multiplexed genome editing as a tool to enhance cellular therapies

While the previous section focused on engineering strategies aiming at introducing synthetic transgenic payloads to enhance immune cell function, the following section aims to portray precision genome editing tools being employed to disrupt and modulate endogenous immune cell pathways which ultimately restrain anti-tumor potency.

### 3.1 Evolution of gene editingtool kits

#### 3.1.1 Nuclease-based genome editing

**Figure 2:**
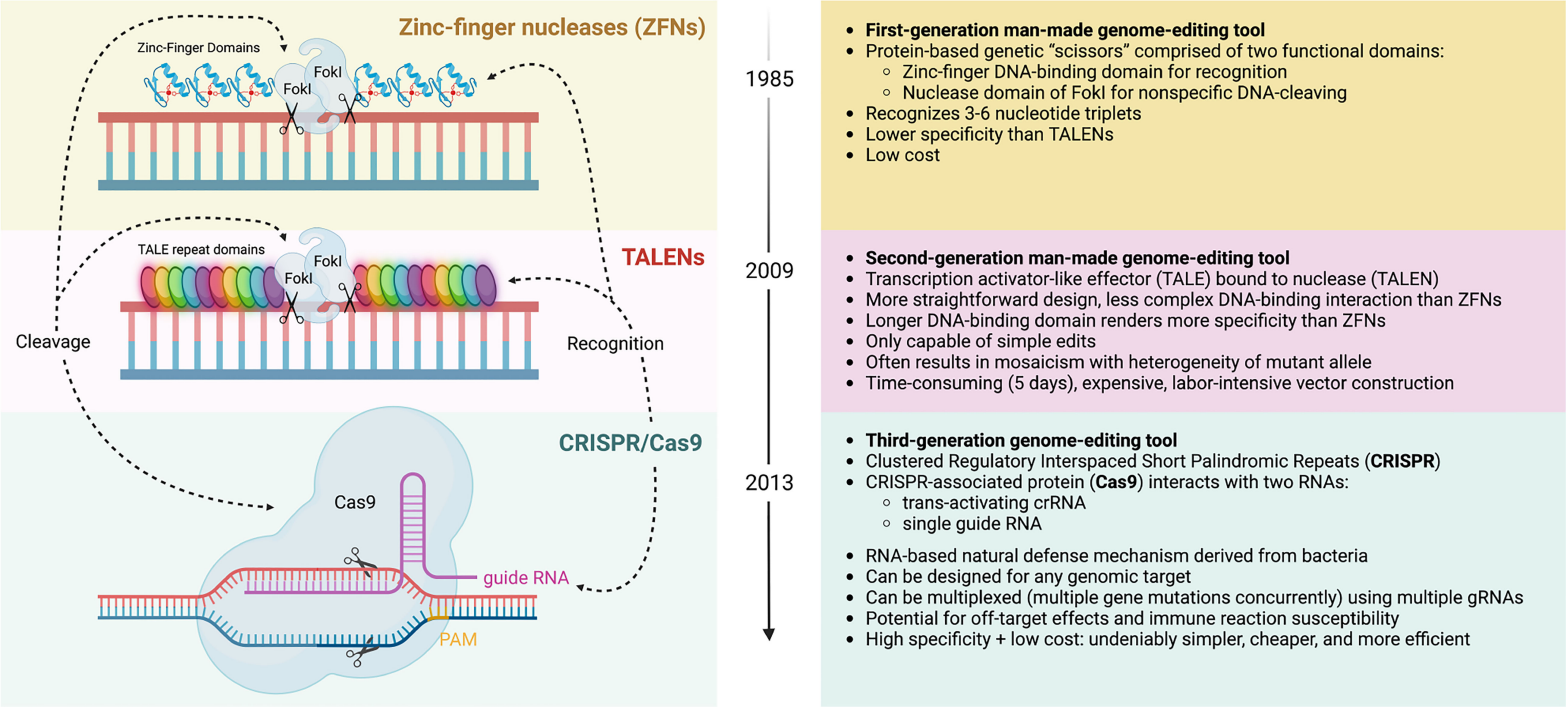
Evolution of gene editing toolkits. Zinc-finger nucleases (ZFNs) are a first-generation man-made gene-editing tool that consists of two domains: 1) a DNA-cleaving domain comprised of the non-specific nuclease domain of FokI to introduce DNA double-stranded breaks, and 2) two DNA-binding domain chains, called “finger” modules, each recognizing a unique hexamer (6 bp) sequence of DNA. The fusion of DNA-binding and DNA-cleaving domains forms a pair of “genomic scissors”, with high specificity but lower than that of Transcription activator-like effector nucleases (TALENs) or Clustered Regulatory Interspaced Short Palindromic Repeats (CRISPR) gene editing systems. TALENs are a second-generation gene-editing tool also based on chimeric nucleases like ZFN with the same non-specific DNA cleavage domain (FokI), but it comprises of longer sequence-specific DNA-binding TALE modules, each of which contacts a single DNA base pair. By fusing with nuclease (TALE-Nuclease or TALEN), this tool can be used to edit genes, but only one at a time, unlike CRISPR. The longer programmable DNA-binding domain comprises of a series of 33-35 amino acid repeat domains, which allows for improved specificity than ZFNs, but increases cost and is marked by a low-efficiency process of vector construction. TALEN editing may induce mosaicism, in which mutations are only present in some transfected cells. The advent of CRISPR/Cas9 gene editing more recently has allowed for the creation of a tool with low cost, high specificity, high efficiency, and much simpler construction. This RNA-based tool is derived from a natural bacterial defense mechanism. The nuclease consists of the CRISPR-associated protein (Cas9) protein classically, though other Cas proteins have been used for cleaving. The complex initially binds to a short sequence known as the protospacer adjacent motif (PAM). This nuclease interacts with two RNAs for recognition and specificity, a trans-activating crRNA and single guide RNA, sometimes combined for simplicity. Notably, CRISPR allows for multiplexed gene editing, where multiple gene mutations can be issued concurrently. Complications include the potential for off-target effects and potential immune reaction susceptibility. As indicated, all systems employ a recognition module and cleavage domain that can be manipulated independently. Created with BioRender.com.

Over the past decades, a plethora of gene editing strategies have been devised to introduce targeted genetic modifications into host genomes of eukaryotic cells (**Figure 2**). Long before the advent of the CRISPR/Cas9 system, zinc-finger nucleases (ZFNs) made their debut in the 1980s and allowed for the first time for site-directed gene editing by recognizing specific genomic sequences, leading to notable successes including the generation of autologous CCR5^-/-^ CD4 T cells for the treatment of HIV (102) and genetic ablation of the checkpoint molecule PD-1 in tumor infiltrating lymphocytes (40). Transcription activator-like effector nucleases (TALENs), which are structurally related to ZFNs, followed in 2009 and further increased target specificity by binding more than 30 base pairs. Within the realm of cell therapy, TALEN technology has been used to engineer allogeneic CAR-T cells which are devoid of their endogenous T cell receptor (TCR) to prevent harmful GvHD (61). Arguably the most prominent toolkit for genetic editing is provided by the CRISPR/Cas9 system, which allows for a high degree of customization with respect to site-specificity at comparably low cost.

**Figure 3:**
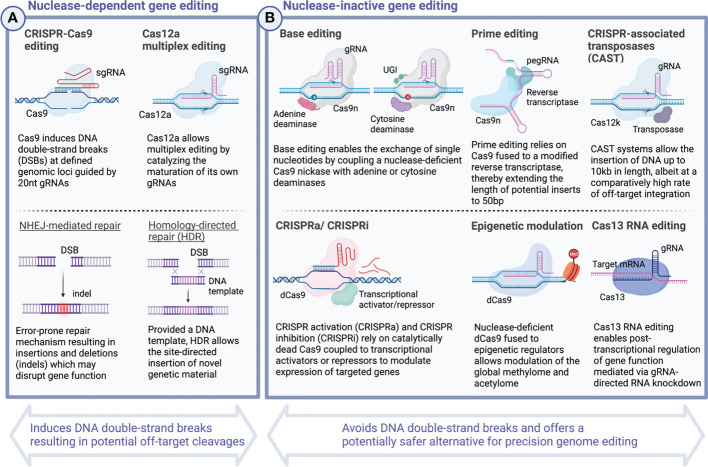
CRISPR - A versatile toolkit for the targeted deletion, rewriting and insertion of novel genetic information. Continuous iterations of technological innovation have considerably extended the scope and versatility of CRISPR-based gene editing strategies. Today, CRISPR genome editing encompasses a wide spectrum of diverse applications including the direct rewriting of genetic code, modulation of gene expression, and post-transcriptional RNA editing. While the most widely known CRISPR-Cas9 system and others rely on inducing DNA double-strand breaks (DSBs) and carry the potential risk of introducing deleterious effects arising from off-target cleavages **(A)**, other CRISPR-based editing tools, including base editing (105, 106) and prime editing (107), allow to rewrite the genetic code without the requirement for DNA double-strand cleavages and, hence, present a potentially safer alternative **(B)**. Over time, the possibilities to insert novel genetic material have expanded dramatically with the most recent advances including Twin prime editing (108) and CRISPR-associated transposase (CAST)-editing (109) now allowing large transgenic inserts in the range of up to 10-40 kb length. Besides editing the genetic code, powerful tools now exist which allow to tune transcriptional activation in a targeted manner. CRISPRa and CRISPRi rely on the site-directed delivery of transcriptional effector molecules to activate or repress gene expression (110–113). More profound epigenetic modulation can be achieved by tethering catalytically dead Cas9 to methyltransferases and acetyltransferases to alter the global methylome and edit histone modifications (114–124). Created with BioRender.com.

Over the past several years, multiple studies have explored preclinical applications of CRISPR technology to optimize cellular therapeutics based on T cells (5–10) as well as NK cells (11–13). In 2020, this progress culminated in the first successful clinical application of CRISPR-engineered TCR-T cells (103). Common to all these Cas9-mediated editing strategies is the induction of double-strand breaks (DSBs) in DNA which are subsequently repaired by either non-homologous end joining (NHEJ) or homology-directed repair (HDR). Incomplete repair of these breakage points may give rise to indels leading to frameshift mutations and subsequent knock-out of one or more targeted genes. When combined with DNA donor template, the same toolkit allows the targeted integration of exogenous DNA inserts at defined genomic loci (104) (**Figure 3**). Incremental improvements have, over time, expanded the potential length of the genetic payloads which can be inserted and increased editing efficiency. Nevertheless, challenges to deliver template DNA to target cells persist and limited abundance of template DNA continues to represent a potential bottleneck for targeted genomic insertions (125–127).

Retron-derived template DNA promise to overcome this technical limitation by drastically increasing the concentrations of available template DNA which in turn may increase precision genome-editing efficiency (128). Retrons serve a role in bacterial phage defense and possess the capacity for reverse transcription (RT) (128–132). Recently, their ability to generate designer RT-DNA *in vivo* was shown to be feasible for human genome-editing applications (128). Over time, it will be interesting to observe how the various platforms of precise-genome engineering will evolve and which ones will prove to be most suitable for clinical translation in the space of engineered immune cell therapeutics.

#### 3.1.2 Nuclease-inactive precision genome editing

Despite their great promises, all previously outlined nuclease-based gene editing strategies hold the potential safety concern of introducing inadvertent off-target cleavages which may lead to potentially deleterious consequences (133–137).

In 2016, researchers around David Liu devised a novel strategy which allows the introduction of genetic alterations without the requirement of inducing DSBs (**Figure 3**). Base editing traditionally refers to the sgRNA-directed exchange of single nucleotides mediated by modified forms of Cas9 protein, Cas9 nickases (nCas9), which lack the capacity to cleave DNA, but instead are fused to bacterial deaminases to substitute single nucleotides (105, 106, 138), although recently CRISPR-free all-protein base editors have been described (139). For nCas9-mediated base editing, single-strand DNA point mutations are subsequently resolved in the process of replication and are used to deliberately alter the codon sequence including insertion of premature STOP codons. Base editors allow correction of pathogenic allele variants offering novel treatment avenues for monogenetic disorders including some causes of sickle cell disease (140) and β-thalassemia (141, 142). The first base editor to have entered the clinic aims to silence *PCSK9* in patients with heterozygous familial hypercholesterolemia and further candidates are already on the horizon (141, 143).

The last years have seen the first applications of base editing to cellular immunotherapy (144), for instance to engineer fratricide-resistant CAR-T cells by targeting the pan-T lineage antigens CD3 and CD7 (145). Another group recently reported the introduction of four simultaneous base edits to generate allogeneic CD7-directed CAR-T devoid of endogenous expression of TRAC, CD52, CD7 and PD-1 (146).

While base editing allows for transition mutations (purine-for-purine and pyrimidine-for-pyrimidine substitutions), it is limited in its ability to perform transversion mutations (exchanging purines for pyrimidines and vice versa), targeted deletions and insertions which would be required to correct for genetic disorders including Tay-Sachs disease and cystic fibrosis among others (107). Prime editing overcomes this limitation by linking nCas9 to a reverse transcriptase, both of which are guided to a desired genomic locus by a customizable prime editing guide RNA (pegRNA) (107) (**Figure 3**). After binding to the target region, prime editing allows for single base exchanges as well as insertions and deletions of synthetic DNA sequences of limited length (107). Efforts are ongoing to increase both prime editing efficiency (147) as well as the potential size of targeted inserts, the latter of which has recently been expanded to incorporate DNA sequences of up to 5kb length using a platform dubbed twin prime editing (TwinPE) (108).

In recent years, the field has seen continuous incremental improvements in editing efficiency, precision, and versatility. Novel genome engineering strategies have emerged which build on existing platforms to further expand their utility. *Programmable Addition via Site-specific Targeting Elements* (PASTE), for instance, uses prime editing technology to insert specific landing sites (AttB) into the host genome which allows subsequent introduction of large genomic sequences of up to 36kb length (148). Yet another strategy for large genomic insertions relies on CRISPR-associated transposases (CAST), which have been demonstrated to enable the insertion of templates of up to 10 kb, however, with a considerable fraction of 50% off-target integrations (109). Against this backdrop, it will be interesting to see how these approaches can eventually be applied to enhance the safety and potency of cell therapy products.

### 3.2 Disruption of immune checkpoints and TME-sensing receptors

Precision genome editing strategies including CRISPR/Cas9 and TALEN allow the disruption of inhibitory immune cell pathways, for instance, by ablation of immune checkpoint molecules or TME-sensing receptors (**Figure 1D**). To enhance CAR-T potency, genetic perturbations have targeted negative regulators of T cell function including PD-1 (41–46), CTLA-4 (43, 45), TIGIT (45), TIM-3 (45–47), LAG-3 (45, 46, 48). Knockdown of GM-CSF in CD19-directed CAR-T cells additionally led to mitigated IEC-mediated toxicities in one pre-clinical study (149). In NK cells, seminal work established the crucial role of the intracellular regulatory checkpoint CIS which acts to suppress cytokine signaling and can be targeted using the CRISPR/Cas9 editing for enhanced NK cellular fitness and potency in non-engineered or CAR engineered settings (49, 50, 150). NKG2A, another potent inhibitory molecule in NK cell, similarly leads to enhanced NK cell potency when genetically blocked, and it will be interesting to see whether this strategy can be translated to build NKG2A-disrupted CAR-NK cells that are resistant to tumors expressing its cognate ligand HLA-E (51).

Receptors sensing the immunosuppressive metabolites of the tumor microenvironment (TME), have likewise been targeted to render NK cells resistant towards these inhibitory cues. Transforming growth factor β1 (TGF-β) is a powerful immunosuppressive cytokine which can have profound negative effects on the anti-tumor immunity of NK cells. CRISPR-mediated targeted ablation of TGF-βR2 rescued NK cell effector functions from these deleterious effects (52, 53). Similarly, genetic disruption of the adenosine A2A receptor may contribute to enhanced CAR-T efficacy as it effectively shields them from the negative consequences of the immunosuppressive metabolite adenosine (54).

### 3.3 Disruption of lineage-specific antigens to mitigate fratricide and expand clinical utility

Other applications using genetic ablation have aimed to delete lineage-specific antigens which might otherwise result in fratricidal killing of engineered immune cells (**Figure 1E**). For CAR-T cells, this has been of particular importance when targeting T cell malignancies which share the same pan-T cell antigens including CD5 and CD7 (57). In NK cells, disruption of endogenous CD38 has enabled the design of cell products which, when combined with anti-CD38 monoclonal antibodies (mAbs), confer anti-myeloma specificity for antibody-dependent cellular cytotoxicity (ADCC) while shielding NK cells from CD38-mediated fratricide (151).

Other manipulations can expand the therapeutic breadth of immune effector cells, e.g., by knocking out CD52 (61, 152) or the glucocorticoid receptor (62) to promote resistance towards treatment-induced immunosuppression when co-administered with alemtuzumab or corticosteroids, respectively (**Figure 1F**).

Furthermore, engineered immune cells can be reprogrammed for more favorable interactions with the host immune system, e.g., by disrupting MHC-1 molecules to avoid host-mediated rejection of engineered T cells, or to prevent GvHD by deleting the endogenous TRAC locus, as is necessary for universal allogeneic CAR-T cell candidates (44, 59, 153).

### 3.4 Sequence-specific editing to enhance immune cell potency

Iterations of TRAC locus engineering now allow for the site-directed insertion of CAR molecules to replace the endogenous T cell receptor signaling complex and thereby reduce the propensity of engineered cells for tonic signaling which has been implicated in premature T cell exhaustion (59). Other strategies leverage sequence-specific editing to fine-tune and optimize antigen sensitivity of engineered T cells as was demonstrated by the generation of HLA-independent T cell receptors (HIT receptors), which exhibit drastically heightened antigen affinity for killing of tumor cells with low antigen density (60).

In conclusion, the high degree of modularity and flexibility for customization provided by the evolving genome engineering technologies has ushered in a new era of cell therapy products which are characterized by rational design approaches building on the ever deeper molecular understandings of tumor-immune cell interactions.

## 4 CRISPR-enabled forward genetic screening

Great strides have been made in recent years to endow immune cells not only with antigen specificity but also enhanced functionality and increased potency by multiplexed genetic engineering and gene editing strategies. Common to all these efforts is that they attempted to overcome previously established and recognized mechanisms of immune cell dysfunction and anergy. While the field is eagerly waiting to see the outcomes when these multi-engineered cell products eventually advance to the clinics, novel unbiased and large-scale discovery approaches have in recent years opened up new avenues to decode novel and undescribed mechanisms of immune cell regulation that might prove instrumental in designing the next generation of engineered immune effector cells.

Pooled loss-of-function CRISPR screens in cancer cell lines have become an invaluable tool to discern molecular mechanisms of therapeutic sensitivity and resistance. A large body of work across a number of different human cancer types has elucidated genetic dependencies that determine responses to anti-neoplastic treatments (154, 155). In recent years, combinatorial screens have further expanded these possibilities and allowed to identify systematically distinct genetic interactions, for instance, to discover synthetic lethal partners which might be exploited for pharmacological interventions. The unique molecular features of the DNA endonuclease Cas12a, which can simplify multiple simultaneous genetic edits, has greatly improved the editing efficiency compared to traditional Cas9 approaches and now permits successful identification of hits in combinatorial drop-out screens (156–159).

Whereas combinatorial screens can bring to light genetic interactions which may drive the onset, maintenance or progression of tumor cells, other platforms set out to investigate cancer-type dependent genetic dependencies. As is increasingly understood, CAR-T resistance mechanisms may vary dramatically across tumor types and explain some of the observed differential sensitivity for solid tumors versus hematologic malignancies (160). Novel platforms aim to investigate these distinct patterns of tumor-intrinsic resistance pathways in a massively parallel manner. For instance, CRISPR loss-of-function screening applied to a pool of hundreds of barcoded cancer cell lines (PRISM) in an orthogonal screening format recently helped elucidate shared cancer-intrinsic transcriptional signatures which correlate with NK cell-sensitivity (161). Noteworthy, this form of therapeutic sensitivity mapping may guide future biomarker-driven treatment algorithms.

### 4.1 Loss-of-function screens in primary immune cells

**Figure 4:**
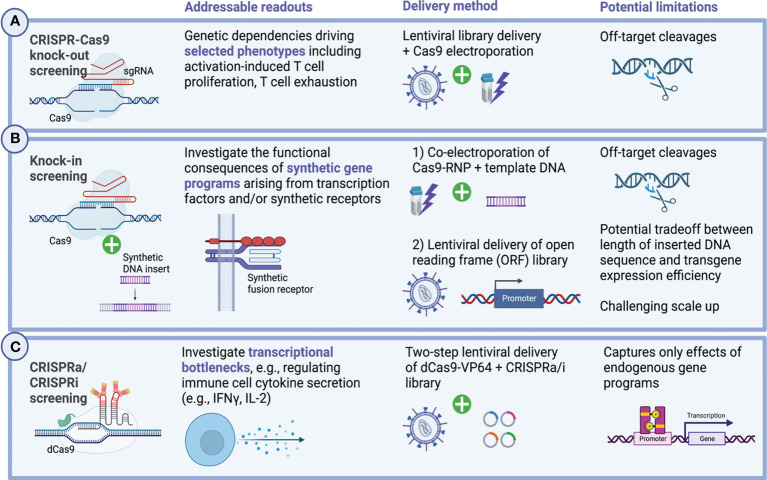
Genome-wide CRISPR screening in primary human immune cells. Forward genetic screening enables the unbiased discovery of genetic dependencies governing cellular functions. Applied to the context of primary immune cells, including genetically engineered immune effector cells, these large-scale discovery approaches have in recent years contributed greatly to develop a deeper understanding of the molecular mechanisms regulating activation-induced T cell proliferation, cytokine secretion and functional exhaustion. Technically, a set of different design approaches exist and enable to address distinct biological questions and confer relationships between genotype and phenotype. **(A)** Pooled CRISPR/Cas9 knock-out screens allow to interrogate in a massively parallel manner the functional consequences of genetic deletions at genome-scale. In primary human T cells as well as CAR-T cells, lentiviral gRNA library delivery is typically followed by transient Cas9 delivery *via* electroporation (162–165). Multiple different readouts have previously been investigated including activation-induced T cell proliferation and cytokine secretion as well as T cell persistence and exhaustion. More sophisticated assays combine these efforts with additional selective pressures, for instance, by challenging T cells with immunosuppressive metabolites such as adenosine to select for biologically relevant phenotypes which might inform the design of the next generation of cellular therapies (162–165). **(B)** Knock-in screens allow the unbiased investigation of novel synthetic gene programs and have lately been translated to primary human T cells in which context they were able to elucidate synthetic fusions receptors which render T cells resistant to immunosuppressive cues from the TME (55, 166, 167). Delivering gene inserts can be accomplished using different strategies, the most recent of which can deliver large transgenes up to multiple kb in size at scale (108, 109). **(C)** Transcriptional remodeling using CRISPRa and CRISPRi-mediated has successfully been applied to primary human T cells and helped elucidate genetic mechanisms orchestrating cytokine secretion of CD4^+^ and CD8^+^ T cells (168). Created with BioRender.com.

Beginning in 2018, CRISPR screening strategies, which sought to directly interrogate different subsets of primary immune cells, started to emerge (**Figure 4**). Previously, technical hurdles in delivering the high-molecular weight Cas9 protein had hampered the efforts of performing such studies in hard-to-transduce primary cells. For this reason, researchers have in many cases leveraged transgenic murine models with constitutive Cas9 expression, circumventing the need to deliver large bacterial endonucleases into primary human immune cells (169–175). Notably, these works have been able to decipher fundamental aspects of immune cell regulation in an unbiased and massively parallel large-scale fashion, complementing decades of research on immunoregulatory pathways governing T cell function.

Genome-wide CRISPR knockout screens in primary human T cells were first reported in 2018, when two groups pioneered a hybrid gene editing approach combining lentiviral sgRNA delivery with transient Cas9 transfection *via* electroporation (162, 163) (**Figure 4A**). In these studies, the authors were able to identify known and unknown genetic mechanisms governing activation-induced proliferation of primary human CD4+ and CD8+ T cells (162) as well as hematopoietic stem and progenitor cell (HSPC) expansion (163). Building on the same platform, the researchers were furthermore able to assess the ramifications of a set of different immunosuppressive selective pressures on immune effector cells (including adenosine, TGF-β, cyclosporine, tacrolimus) to identify potential routes to eventually overcome them (162, 164).

#### 4.1.1 High-content CRISPR readouts

Another aspect, which has greatly improved the biological relevance of these studies is driven by the advent of high-content CRISPR readouts. By relying on, for instance, marker-based sorting to differentiate distinct cellular phenotypes or by integrating other high-dimensional assays including single cell RNA sequencing, these next-generation CRISPR screens provide more depth compared to traditional assays which primarily addressed resistance or sensitivity to a certain external selective pressures (176).

One prominent example comes from work done by the group of Jeremy Rich, who investigated genetic determinants of exhaustion, as defined by surface expression of PD-1, in EGFRvIII-directed CAR-T cells when challenged with glioblastoma stem cells (GSCs) (165). In their seminal work, they used cell sorting to assess guide enrichment in PD-1^+^ CAR-T compartment and found crucial regulators of CAR-mediated glioblastoma killing. RNA sequencing of GSC-challenged CAR-T cells, which had been specifically edited based on previously identified hits, revealed distinct transcriptomic signatures characterized by upregulation of T cell activation markers and proinflammatory cytokines.

The integration of CRISPR screening and single cell RNA sequencing, hence, powerfully illustrates the potential of high-dimensional studies to capture the complex regulatory mechanisms which determine immune cell fates and anti-tumor immunity (162, 165). In light of the recent report of the first genome-scale mapping of transcriptional effects of genetic perturbations (Perturb-seq) (177), going forward, the relevance of these information-rich assays is likely going to further increase and will help inform the rational design of next-generation engineered cell products.

### 4.2 Knock-in screening

While targeted genetic ablation has the potential to disrupt pathways which restrain immune cell activation or contribute to exhaustion, knock-in of large gene cassettes, may hold even greater potential to reprogram immune cells for heightened effector functions or exhaustion resistance (32). As has recently been demonstrated, forward genetic screening can be deployed to systematically investigate the transcriptional and functional ramifications arising from the introduction of synthetic gene programs in pooled gain-of-function screens in primary human T cells (55, 166, 167) (**Figure 4B**). Two main technical platforms for gain-of-function screening have been described relying on either i) non-viral delivery of promoterless double-stranded DNA templates to a specific locus *via* co-electroporation with CRISPR/Cas9 ribonucleoproteins (RNPs) for subsequent HDR-mediated integration (55) or ii) lentiviral delivery of a barcoded open reading frame (ORF) library (166). Of note, these platforms allow to systematically interrogate the functional consequences of the integration of completely novel gene programs including synthetic receptors which physiologically might not be present in the targeted immune cells, a key difference compared to CRISPR activation (CRISPRa)-based screens, which focus on activation of endogenous genes (168, 178–180). For instance, insertion of lymphotoxin-β receptor (LTBR), typically expressed in myeloid cells, into unmodified as well as CAR-engineered T cells, led to exhaustion resistance and improved effector functions by formation of an autocrine positive feedback loop, as a recent genome-scale pooled knock-in screen selecting for highly-dividing primary T cells revealed (166). Similarly, an *in vivo* pooled knock-in screen which assessed the relative genotype abundance of 36 synthetic receptors, transcription factors and metabolic regulators among melanoma-infiltrating lymphocytes (TILs), found that introduction of a novel chimeric TGF-β-R2-41BB receptor endowed T cells with the capacity to resist immunosuppressive signaling from TGF-β leading to enhanced melanoma clearance *in vivo* (55). As the field evolves, both the breadth of screenable gene products has increased and the associated sequencing efforts have been streamlined using modular screening strategies (167). These modular platforms also enable combinatorial screening, for instance, to investigate the synergies from the combined integrations of different transcription factors (167).

Collectively, these examples demonstrate the power of CRISPR-based unbiased discovery tools to identify novel gene programs, which, when introduced into engineered lymphocytes might augment cellular fitness and anti-tumor potency.

### 4.3 Tuning transcriptional networks for enhanced immune cell function

Today, CRISPR-mediated genome editing allows for the targeted ablation, rewriting and introduction of entirely new segments of DNA (105–107, 110–113, 181). Specific modifications using a catalytically dead form of Cas9 (dCas9) fused with transcriptional activator (CRISPR activation – CRISPRa) or repressor molecules (CRISPR interference – CRISPRi) furthermore allow the targeted epigenetic modulation of gene expression (110–113, 123, 124) (**Figure 3**). Both CRISPRi and CRISPRa have been used to screen for transcriptional networks regulating cellular function at genome-scale (177, 180, 181) to decipher resistance mechanisms involved in dampening anti-tumor immunity (180). Recent efforts point to the potential application of multiplexed CRISPRa technology as a tumor vaccine by upregulating TAAs for enhanced T cell anti-tumor immunity (179).

In primary human T cells, reciprocal genome-wide CRISPRi/CRISPRa screening has been used to expose transcriptional bottlenecks which restrict T cell activation. Paired with scRNAseq (Perturb-seq), the study further enabled deep molecular characterization of the identified therapeutically relevant T cell states which may prove instrumental when designing novel T cell-based immunotherapies (168) (**Figure 4C**). In another recent study, CRISPRa gain-of-function screening was applied to CAR-modified murine T cells and was able to demarcate proline metabolism as a pivotal driver of CAR-T cellular fitness and function (178).

Of note, the CRISPRa toolkit is also being leveraged by adjacent research fields, for instance, to revisit fundamental questions of tumor biology, such as whether specific mutations implicated in tumor resistance have been acquired through clonal evolution or were pre-existing. Such questions are now possible to answer using CRISPRa-inducible reporters for live clonal retrieval (182).

#### 4.3.1 Transcriptome editing using CRISPR-Cas13 proteins

Whereas CRISPRi and CRISPRa rely on the stable introduction of transcriptional activators and suppressors to modulate gene expression, Cas13 allows for direct transcriptome engineering *via* RNA editing without the requirement for permanent genetic manipulations (183–186) (**Figure 3**). CRISPR-Cas13-mediated transcriptome engineering now allows for both targeted RNA knockdown as well as the rewriting of RNA transcripts, e.g., to correct for pathogenic mutations (187). Of note, Cas13-based approaches may suffer from some of the same drawbacks and limitations as Cas9 including potential off-target editing (188) and host rejection due to pre-existing immunogenicity (189). Nevertheless, this emerging technology represents a promising toolkit for transcriptome engineering which is potentially safer than CRISPR-Cas9-mediated approaches as it can induce defined cellular states without introducing genomic alterations. Recent innovations further yielded chemically modified forms of Cas13, which were able to stably modulate gene expression in CD4 and CD8 T cells (190).

The potential implications of these next-generation CRISPR tools are vast and aim to i) better understand the basic biology of anti-tumor immunity and ii) to build better and more powerful cellular therapeutics. Moreover, while a number of potential candidate genes for augmented T cell immunotherapy have been proposed, it will be critical to see how robustly pre-clinical validation efforts of identified hits can predict the successful transition into clinical development programs.

## 5 *In vivo* gene editing and cell engineering strategies

In its latest iteration, precision genome engineering has been adapted for cell-type specific *in vivo* genetic editing to treat various human diseases including cancer. In a report from 2021, CRISPR/Cas9-mediated *in vivo* deletion of transthyretin (*TTR)*, which is mutated in progressively fatal transthyretin amyloidosis, led to a striking decrease of circulating transthyretin protein levels in the peripheral blood of treated patients (191). Using an apolipoprotein-E (ApoE)-recruiting lipid nanoparticle (LNP) loaded with Cas9-encoding mRNA and a *TTR*-directed sgRNA, this platform allows to specifically correct this pathogenic variant in hepatocytes (192). Similarly, pre-clinical efforts have resulted in the *in vivo* generation of fibroblast activation protein (FAP)-directed CAR-T cells to treat cardiac injury, by selectively reprogramming T cells using a CD5-targeted LNP system (193). Besides LNP-mediated strategies, implantable bioinstructive scaffolds have been developed, which can generate CAR-T cells *in vivo* and, hence, circumvent the cost-intensive and lengthy vein-to-vein times required for *ex vivo* manufacturing of autologous CAR-T products (194).

Most recently, nuclease-inactive genome editing strategies have also transitioned to *in vivo* applications to correct pathogenic mutations in diseases like Hutchinson–Gilford progeria syndrome (195) or familial hypercholesterinemia using size-optimized adeno-associated viruses (AAV) as delivery vehicles (196).

These developments may have far-reaching implications, as they promise to dramatically reduce the time and cost of manufacturing cell products, which could ultimately increase patient access. Furthermore, the immense adaptability of precision genome editing strategies for designing and delivering DNA sequences to correct pathogenic allele variants make it likely that these powerful tools will play an increasingly important role in the treatment of many of the most devastating human diseases.

## 6 Ensuring patient safety and navigating regulatory challenges

Despite the well-founded optimism, safety concerns arising from various engineering and genome editing strategies remain a preeminent concern, particularly when novel multiplex modified cellular therapeutics transition into the clinic. Recently, temporary halts of ongoing clinical trials investigating gene-edited cell products illustrated the delicate regulatory trade-off to find the right balance between granting patients access to innovative treatments while ensuring utmost patient safety.

Safety concerns may arise both from the vector system employed to deliver genetic cargo or from the induced genetic alterations, themselves. While overall vector safety has greatly improved over the last decades, nuclease-based genome editing strategies continue to carry the risk of inadvertent off-target cleavages. Off-target toxicities of CRISPR/Cas9 editing include mutational events or chromosomal rearrangements that may appear despite the first 20 nucleotide sequence of the sgRNA directing the efficiency of Cas9. Inadvertent off-target cleavage may lead to the deleterious loss of tumor suppressor genes (197). One such example of off-target mutations is the integration of DNA mismatches in the PAM-distal portion of the sgRNA sequence (134, 135, 198–200). Multiple unbiased sequencing platforms exist today and allow to detect these undesired genomic edits (201–203) and assess potential associated risks to decide whether a particular cell product can be safely infused. Global and sensitive detection of DSBs introduced by CRISPR RNA-guided nucleases is enabled through Genome-Wide, Unbiased Identification of DSBs enabled by Sequencing (GUIDE-seq) (201), Circularization for *in vitro* Reporting of Cleavage Effects by Sequencing (CIRCLE-seq) (202), or RNase H-dependent PCR Amplification Sequencing (rhamp-Seq) (203), which all function as assays to assess off-target cleavage. Approaches to diminish or prevent off-target effects of CRISPR/Cas9 editing include (i) identifying unique target sites without homology to other genomic regions, (ii) modifying the sgRNA by distal truncation of the 3’ end (204), (iii) substitution of regular Cas9 with high fidelity Cas9 (HiFi) or alternative nucleases such as Cas12 (205), (iv) lowering the concentration and optimizing the ratio of Cas9-sgRNA given to the cells (206), or (v) fusing dCas9 with Fokl nuclease (fCas9) to allow for higher sequence targeting specificity (207, 208). Alternatively, nuclease-inactive genome engineering including base editing and PRIME editing present potentially safer alternatives as they avoid DNA double-strand breaks and may well evolve to play a more important role for genome-edited cell products for clinical use going forward.

Today, clinical development programs aiming to translate promising cellular candidates into the clinic are in ever closer contact with regulatory bodies to jointly sketch a safe path forward for highly innovative products to enter the clinic. As the field matures, the most relevant learnings have recently been formalized in Food and Drug Administration (FDA)-issued guidance documents. One important document is focused on *Considerations for the Development of Chimeric Antigen Receptor (CAR) Cell Products* (209) and another important document is geared towards *Human Gene Therapy Products Incorporating Human Genome Editing* (210).

Within the framework of these resources, the FDA has issued recommendations regarding the chemistry, manufacturing, and control (CMC), as well as toxicology, pharmacology, and clinical study design involving CAR engineered immune effector cell therapies (209). A prominent guidance involves the choice of vector for gene delivery. Vectors integrating into cellular DNA (e.g., retroviral-based vectors or transposons) may increase the risk of delayed adverse events, including potentially by insertional mutagenesis, and this may require long-term follow-up. Such occurrences would be unexpected in non-integrating vectors, and thus such long-term follow-up for these products may not be warranted. The FDA advises that the necessity of each additional functional domain be justified in application reports, with a description of how these elements may affect CAR specificity, functionality, safety, and immunogenicity. Batch-based manufacturing and process optimization are recommended. If viral vectors are utilized, transduction reliability with batch-to-batch consistent viral multiplicity of infection (MOI) is critical. Aseptic technique should be carried out under current good manufacturing process (CGMP) conditions. Guidance also covers the manufacture and delivery of CAR products, with allowance decreed to both fresh and cryopreserved products depending on the desired shelf-life and quality of the products. Cryopreserved cell products may be more practical for delivery of the cells to different clinical sites, provided that controlled thawing procedures are carefully outlined, potential transport plans are established, and assurance regarding the safety of cryoprotectants has been assessed. Regarding study design, sponsors are recommended to arrange *a priori* analyses for all subjects, as well as for patients stratified by the receipt of bridging therapy or not. A plan for stopping rules from dose-limiting toxicities, adverse effect monitoring and attribution, and follow-up must be described.

The FDA has outlined separate guidelines for human gene therapy products incorporating human genome editing, encompassing CRISPR-engineered cell products (210). They elaborate on different genome editing tools, delivery methods, and conditions (e.g., *ex vivo*, *in vivo*), with stipulated goals to stabilize components, uphold aseptic processing, and minimize off-target genome alterations. The IND should contain documentation regarding genome editing efficiency and specificity. With appropriate controls, orthogonal and redundant models are recommended that incorporate *in silico*, biochemical, and cellular-based assays, for unbiased genome-wide analysis to detect potential off-target sites. Safety analyses should include assessments of the immunogenicity of genome editing components, viability assessment, evaluation for any residual gene editing components, and testing for selective survival advantage of edited cells, including dysregulated growth or clonal proliferation. It is essential to evaluate genomic integrity, including deletions or insertions, chromosomal rearrangements, exogenous DNA integration, and potential insertional mutagenesis or oncogenicity.

## 7 Concluding remarks and future directions

The intersection of multiplexed engineering, precision genome editing, and cellular immunotherapy has brought about an unprecedented wave of innovations which has over the last couple of years dramatically expanded the available treatment modalities to combat cancer and other devastating diseases. Iterative cycles of innovation have extended the scope of genome editing strategies and novel delivery methods have helped enable the design of multiplexed-engineered cell products, redrawing the treatment landscape in oncology and beyond. Nevertheless, important challenges remain, and the field is actively exploring opportunities to overcome them.

First, there is an imperative to better understand the mechanisms of therapeutic resistance and relapse following cellular immunotherapy. Devising strategies to overcome tumor immune escape and prevent functional exhaustion of infused immune cells while mitigating inadvertent toxicities are just two examples of how improved cellular therapeutics may evolve to become more powerful with time. As outlined, forward genetic screening approaches paired with information-rich multi-omic analyses may critically inform these novel strategies by uncovering the biological mechanisms which orchestrate the complex interplay between immune cells and tumors. Multiplexed gene engineering may, in turn, build on these learnings to enable the design of more powerful cell products which can produce more durable and potent anti-tumor immune responses.

Sketching a path forward for cellular immunotherapy to effectively combat solid tumors indisputably represents one of the most formidable challenges, the field seeks to address, and numerous highly differentiated strategies are being pursued to accomplish this goal. Besides tackling the challenges of shared antigen expression (35–37, 211), downregulation of MHC molecules (212–214) and impaired trafficking (39), the field is continuously branching out, for instance, towards aiming to restore the immunogenicity of immunologically ‘cold’ tumors, *via* various approached including epigenetic modulation (215, 216), tumor vaccination strategies (179) or by incorporating bacterial virulence factors to attract bystander immune cells (217). Researchers have also investigated the possibility of delivering CAR targets to solid tumors *via* oncolytic viruses, in the hope to render the transformed cells targetable using established CAR-T cell products (218). Furthermore, recent work has also pointed to other immune cell subsets for CAR modification (1, 219–223), including CAR-modified macrophages (CAR-M), which may provide unique advantages to better penetrate solid tumor capsules (224). Following the recent report of *in vivo* edited hepatocytes (191), future studies may also aim to reprogram the tumor cells directly *in vivo*. This approach might be leveraged to restore treatment sensitivity by augmenting the release of immune cell-attracting chemokines (99, 225) or by forcing expression of TAAs for enhanced immunogenicity (179). Other strategies might aim to selectively induce cell death, for instance, by targeted disruption of oncogenes or hybrid pharmaco-genetic interventions to suppress synthetic lethal gene interactions.

Lastly, a growing number of stakeholders in the field are aiming to expand the scope of eligible patients who can benefit from the tremendous advancements of cellular immunotherapy and precision genome engineering. The transition towards allogeneic off-the-shelf cell products (16, 31, 73, 226, 227) and *in vivo*-manufactured cell products (193, 194) is already in full swing. Bi- and multi-specific engager molecules are being explored to endow immune cells with CAR-like anti-tumor specificity (228–237), bypassing the complexities associated with cellular engineering entirely. Combinations of both approaches in the form of BiTE-secreting CAR-T cells epitomize the highly modular nature of next generation cellular immunotherapy (238).

Going forward, we expect that these and other emerging technologies will further advance and deepen our understanding of cancer biology and help engineer better, more potent and safer immunotherapies to combat various human cancers.

## Author contributions

AB and MD conceived the outline for this manuscript. AB, GM, and MD wrote the manuscript and created figures. All authors contributed to the article and approved the submitted version.

## Funding

AB received support from the German Research Foundation as Walter Benjamin Postdoctoral Fellow (464778766). GM receives support from the Radiological Society of North America (RSNA) and the American Society for Radiation Oncology (ASTRO). The project described was supported by RSNA Research & Education Foundation, through the Canon Medical Systems, USA/RSNA Research Resident Grant. The content is solely the responsibility of the authors and does not necessarily represent the official views of the RSNA R&E Foundation. MD acknowledges support from the Society for Immunotherapy of Cancer (SITC), The Leukemia SPORE developmental research program award, the Andrew Sabin Family Foundation and the MD Anderson AML and B-cell lymphoma moonshot programs.

## Conflict of interest

MD and The University of Texas MD Anderson Cancer Center have an institutional financial conflict of interest with Takeda Pharmaceutical for the licensing of the CAR-NK cell technology.

The remaining authors declare that the research was conducted in the absence of any commercial or financial relationships that could be construed as a potential conflict of interest.

## Publisher’s note

All claims expressed in this article are solely those of the authors and do not necessarily represent those of their affiliated organizations, or those of the publisher, the editors and the reviewers. Any product that may be evaluated in this article, or claim that may be made by its manufacturer, is not guaranteed or endorsed by the publisher.
